# Theories of behaviour change synthesised into a set of theoretical groupings: introducing a thematic series on the theoretical domains framework

**DOI:** 10.1186/1748-5908-7-35

**Published:** 2012-04-24

**Authors:** Jill J Francis, Denise O’Connor, Janet Curran

**Affiliations:** 1 Health Psychology Group and Health Services Research Unit, University of Aberdeen, Aberdeen, UK; 2 School of Public Health and Preventive Medicine, Monash University, Victoria, Australia; 3 Clinical Epidemiology Program, Ottawa Hospital Research Institute, University of Ottawa, Ottawa, Canada

## Abstract

Behaviour change is key to increasing the uptake of evidence into healthcare practice. Designing behaviour-change interventions first requires problem analysis, ideally informed by theory. Yet the large number of partly overlapping theories of behaviour makes it difficult to select the most appropriate theory. The need for an overarching theoretical framework of behaviour change was addressed in research in which 128 explanatory constructs from 33 theories of behaviour were identified and grouped. The resulting Theoretical Domains Framework (TDF) appears to be a helpful basis for investigating implementation problems. Research groups in several countries have conducted TDF-based studies. It seems timely to bring together the experience of these teams in a thematic series to demonstrate further applications and to report key developments. This overview article describes the TDF, provides a brief critique of the framework, and introduces this thematic series.

In a brief review to assess the extent of TDF-based research, we identified 133 papers that cite the framework. Of these, 17 used the TDF as the basis for empirical studies to explore health professionals’ behaviour. The identified papers provide evidence of the impact of the TDF on implementation research. Two major strengths of the framework are its theoretical coverage and its capacity to elicit beliefs that could signify key mediators of behaviour change. The TDF provides a useful conceptual basis for assessing implementation problems, designing interventions to enhance healthcare practice, and understanding behaviour-change processes. We discuss limitations and research challenges and introduce papers in this series.

## Background

Behaviour change is key to increasing the uptake of evidence into healthcare practice. Behavioural science in general, and health psychology in particular, abounds with plausible, evidence-based theories and models that purport to explain and predict behaviour and behaviour change. It makes sense to design interventions on the basis of such models. However, to the multidisciplinary implementation research community and, often, to health psychologists as well, there is a bewildering array of theories from which to choose. Selecting one theory or a few theories as the basis for intervention design leaves the researcher (or reviewer) in doubt as to whether some key factor may have been omitted. The need for an overarching theoretical framework has been addressed in an influential line of research in which 128 explanatory constructs from 33 theories of behaviour were identified [1]. Key constructs relevant to changing the behaviour of healthcare professionals were grouped into 12 ‘theoretical construct domains’. The 12 domains are labelled (1) Knowledge; (2) Skills; (3) Social/Professional Role and Identity; (4) Beliefs about Capabilities; (5) Beliefs about Consequences; (6) Motivation and Goals; (7) Memory, Attention, and Decision Processes; (8) Environmental Context and Resources; (9) Social Influences; (10) Emotion; (11) Behavioural Regulation; and (12) Nature of the Behaviours. The resulting Theoretical Domains Framework (TDF) has been used in a variety of contexts to inform and address implementation problems.

Interview questions and questionnaire items may be designed to explore the specific content of these domains in relation to implementation problems. The TDF may also be used as a coding framework for analysis. The theoretical domains are proposed to be potential mediators of behaviour change (except for Nature of the Behaviours, which is accorded a different status to the rest, as it relates to the essential characteristics of the behaviour of interest rather than possible mediating mechanisms or influences on behaviour). Each domain consists of a grouping of theoretical constructs (where constructs are defined as component parts of theories, such as ‘attitude’, ‘self-efficacy’, ‘anxiety’). For example, the domain Social Influences includes such constructs as social support, group norms, group conformity, social pressure, social comparisons, and several others [1]. In this example domain, the pertinent constructs are grouped together to represent the influences of people on others’ behaviours.

Further consensus work has identified some of the behaviour-change techniques that are likely to be effective (and others that are likely to be ineffective) if they target specific domains when these domains are identified as likely mediators of change [2]. The 12 theoretical construct domains thus represent a large range of theoretical approaches and can be used for problem analysis, theorising pathways of change, designing interventions, identifying appropriate process measures, and testing pathways to change [1].

### Objectives of the thematic series

At this time, over 6 years after the publication of the original TDF paper [1], it is timely to document the impact of this framework on implementation research and to consider its strengths, limitations, and potential for further use and development. This article provides a brief overview of the TDF and serves as an introduction to a thematic series on the TDF for *Implementation Science*. Research groups from several countries, exploring a variety of implementation problems, have contributed to this series in order to

(1) demonstrate the breadth of behaviours, clinical settings, designs, and methods that have used the TDF;

(2) explain how the TDF can be applied and operationalised to explore implementation problems and design implementation interventions;

(3) describe theoretical and methodological developments based on the TDF;

(4) raise questions that may suggest an agenda for future TDF research.

### Utilisation of the theoretical domains framework in implementation research

To document the impact of the TDF on implementation research to date, we conducted a brief indicative review. We searched the abstracts (and full text, if required, for clarity) of all papers citing the paper that described the development of the TDF [1] to 30 November 2011 (identified through http://www.scopus.com). We selected, for further description, the studies that used the TDF as the basis for an empirical study. We noted the range of journals in which these studies were published, the countries in which the studies were conducted, the behaviours investigated, and the study designs used. Where relevant for identifying impact or validity, we also extracted the specific findings or methods reported in these studies.

Of the 133 citing papers (from 83 scientific journals) indexed in the Scopus database, 23 papers reporting 21 different studies used the TDF as the basis for an empirical study. Where a study protocol and a results paper were published with respect to the same study, we included only the results paper. Of the 21 studies identified, 17 investigated the behaviour of health professionals and four investigated health-related behaviour of members of the public. Ten of the 21 studies described exploratory interview studies (with individuals or focus groups) designed to identify barriers and levers to uptake of a guideline [3-13], often to inform intervention design. Two reports described questionnaire studies [13,14], and two reported both interviews and questionnaires [15,16]. There were four systematic reviews in which the theoretical domains were investigated as mediators of behaviour change [17-20], two randomised studies [21,22], and one protocol for a process evaluation study to explain trial effects in the context of a randomised trial [23]. The included studies provide evidence that the TDF has considerable breadth and cross-disciplinary impact in research about health-related behaviour (studies were published in 13 journals) and geographical reach (six countries from four continents were represented). Table1 presents a summary of the characteristics of studies that have used the TDF to investigate health behaviours.

**Table 1:** Summary characteristics of studies citing the original Theoretical Domains Framework (TDF) paper that used the framework in an empirical study

**Lead author, source, date**	**Paper title**	**Study design of TDF-based component**	**Sample**	**Behaviour; setting**
Jacobs N. *Patient Education & Counseling* 2011:85(1);122	Effect of a cardiovascular prevention program on health behaviour and BMI in highly educated adults: A randomized controlled trial	Randomised controlled trial	Highly educated adults (n = 314)	Fat intake, physical activity, smoking; Belgium
Amemori M. *Implementation Science* 2011:6(1); 50	Assessing implementation difficulties in tobacco use prevention and cessation counselling among dental providers	Questionnaire study	Dental healthcare providers (n = 73)	Providing tobacco use prevention techniques and cessation counselling; Finland
Zhu D. *Obesity Reviews* 2011:12(501);e324	The relationship between health professionals’ weight status and attitudes towards weight management: A systematic review	Systematic review	Health professionals from 14 independent samples (n = 10,043)	Providing weight management advice
Helms C. *Vaccine* 2011:29(16);2895	Implementation of mandatory immunisation of healthcare workers: Observations from New South Wales, Australia	Interview study	Stakeholders from health department, hospitals, health professional associations, universities (n = 58)	Immunisation of healthcare workers; Australia
Dyson J. *Journal of Infection Prevention* 2011:12(1);17	Does the use of a theoretical approach tell us more about hand hygiene behaviour? The barriers and levers to hand hygiene	Interviews, focus groups, and questionnaire study	Healthcare practitioners (n = 25, 21, 24 for interviews, focus groups, and questionnaire, respectively)	Hand hygiene behaviours; UK
Ivers NM. *Implementation Science* 2010:5(1); 98	Feedback GAP: Study protocol for a cluster-randomized trial of goal setting and action plans to increase the effectiveness of audit and feedback interventions in primary care	Protocol for process evaluation, involving interviews, to investigate barriers to change in trial context	Primary care practitioners (target n = 12)	Primary care; Canada
Cuthbertson B. *Trials* 2010:11; 117	A study of the perceived risks, benefits and barriers to the use of SDD in adult critical care units (the SuDDICU study)	Protocol for multistage feasibility study involving interviews for a Delphi study	Four stakeholder groups involved in intensive care (critical care, infectious diseases, pharmacy, nursing) (target n = 120)	Provision of Selective Decontamination of the Digestive Tract (SDD) in Critical Care; UK, Canada, Australia, New Zealand
McKenzie JE. *Implementation Science* 2010:5(1); 86	Improving the care for people with acute low-back pain by allied health professionals (the ALIGN trial): A cluster randomised trial protocol	Protocol includes interviews; findings used to design intervention	Physiotherapists and chiropractors in Australia (210 practices)	Behaviours from a clinical practice guideline for acute low back pain; Australia
Hetrick S. *Australasian Psychiatry* 2010:18(5);451	Promoting physical health in youth mental health services: Ensuring routine monitoring of weight and metabolic indices in a first episode psychosis clinic	Interview study	Psychiatrists (n not reported)	Monitoring of weight gain and metabolic indices in people with first episode psychosis; Australia
Brotherton JML. *Sexual Health* 2010:7(3):291	National survey of general practitioners’ experience of delivering the national human papillomavirus vaccination (HPV) program	Questionnaire study	General practitioners (n = 298)	Delivery of HPV vaccine, general practice; Australia
Clarkson JE. *Implementation Science* 2010:5(1);57	The translation research in a dental setting (TRiaDS) programme protocol	Questionnaires and interviews used to develop interventions	Range of samples, dentistry staff	Range of dental care behaviours; UK
Edwards P. *BMC Public Health* 2010:10;200	Assessing the effectiveness and cost-effectiveness of adaptive e-Learning to improve dietary behaviour: Protocol for a systematic review	Systematic review	Participants aged 13 years or over	Dietary behaviour
Guillaimie L. *International Journal of Behavioral Nutrition & Physical Activity* 2010:7;12.	Psychosocial determinants of fruit and vegetable intake in adult population: A systematic review	Systematic review	General population (n = 34,577)	Fruit and vegetable intake
McCluskey A. *BMC Health Services Research* 2010:10;18	Delivering an evidence-based outdoor journey intervention to people with stroke: Barriers and enablers experienced by community rehabilitation teams	Before-after interview study	Allied health professionals from two rehabilitation teams (n = 13)	Delivery of evidence-based outdoor journey intervention for people with stroke; Australia
Nzinga J. *Implementation Science* 2010:4(1); 44	Documenting the experiences of health workers expected to implement guidelines during an intervention study in Kenyan hospitals	Interview study	Health workers (n = 29)	Paediatric and newborn care in hospitals; Kenya
Godin G. *Implementation Science* 2010:3(1); 36	Healthcare professionals’ intentions and behaviours: A systematic review of studies based on social cognitive theories	Systematic review	Studies of health professionals’ behaviour (n = 78)	Studies based on social cognitive theories
Francis JJ. *British Journal of Health Psychology* 2009:14(4);625	Evidence-based selection of theories for designing behaviour change interventions: Using methods based on theoretical construct domains to understand clinicians’ blood transfusion behaviour	Interview study	Intensive care consultants and neonatologists (n = 18)	Intensive care and paediatric intensive care; UK
Judah G. *American Journal of Public Health* 2009:99(Suppl.2)	Experimental pretesting of hand-washing interventions in a natural setting	Naturalistic randomised study; hour of the day as unit of randomisation	General population (n = nearly 200,000 restroom uses)	Hand washing in public restrooms; UK
Pitt VJ. *Disability & Rehabilitation* 2008:30(25);1938	Referral of people with osteoarthritis to self-management programmes: Barriers and enablers identified by general practitioners	Interview study	General practitioners (n = 13)	Referral of people with osteoarthritis to self-management programmes; Australia
McKenzie JE. *Implementation Science* 2008:3(1); 11	IMPLEmenting a clinical practice guideline for acute low back pain evidence-based manageMENT in general practice (IMPLEMENT): Cluster randomised controlled trial study protocol	Protocol includes focus group interviews; findings used to design intervention	General practitioners (target sample size = 92 general practices)	Behaviours from a clinical practice guideline for acute low back pain; Australia
Michie S. *Implementation Science* 2007:2(1); 8	Difficulties implementing a mental health guideline: An exploratory investigation using psychological theory	Interview study	Professionals in community mental health teams (n = 20)	Offering a family intervention to families of people with schizophrenia; UK

*BMI* body mass index; *GAP* goal setting and action plans; *SuDDICU* selective decontamination of the digestive tract in intensive care units; *ALIGN* acute low-back pain: implementing guidelines iNto practice.

Papers excluded from further description were editorials and opinion pieces, empirical studies in which the TDF was used to support an aspect of the rationale, and empirical studies based on other theories in which the TDF was cited in the discussion of the study findings.

## Discussion and critique

The TDF appears to have succeeded in ‘making psychological theory useful’ to researchers from a variety of disciplinary backgrounds internationally, to investigate a wide range of behaviours in various healthcare settings. The great majority of the identified papers report implementation research, and half of the identified papers report interview studies. Critics of the TDF might argue that an interview topic guide based on the framework is too focused and too constraining and would lead participants to select only the views and opinions about the topic that fit into the framework. However, one study used randomised designs to make direct comparisons of results when methods were based on the TDF versus atheoretical methods, using interviews, focus groups, and a questionnaire [16]. Although there was considerable overlap in the findings from the two approaches, the TDF-based studies elicited beliefs that were not mentioned in the studies that had no theoretical basis. Furthermore, the data generated using the TDF approach were more likely to elicit beliefs about the impact of emotional factors on behaviours, demonstrating that the framework does not limit its investigation to rational or ‘cognitive’ processes [16]. This suggests that the theoretical coverage of the TDF is comprehensive and that it can facilitate an inclusive, rather than selective, approach to exploratory research in the field of implementation.

Hence, two major strengths of the TDF are its theoretical coverage and its capacity to elicit a comprehensive set of beliefs that could potentially be mediators of behaviour change. In its current form and as currently used, one limitation is that it is a descriptive framework rather than a theory, that is, it does not specify relationships between the domains [9] and thus does not generate testable hypotheses. Another limitation relates to the fact that the majority of studies to date have used the TDF in interview studies (Table1), which are characterised by the problem that data collected from an interview reflect participants’ attributions about the influences on their behaviours, including possible attributional biases [24,25], and may not necessarily reflect ‘actual’ causes. However, this limitation is a function of the research designs that are frequently used in TDF-based studies rather than a limitation of the framework itself. In the context of interview studies, a further limitation is that inter-coder agreement can be relatively low [9], perhaps reflecting a difficulty that some research teams have in clarifying the boundaries between domains when the TDF is used as a coding framework.

It is perhaps appropriate to offer a word of warning to researchers who wish to base their research on the TDF. The domains integrate theoretical constructs that have been developed during the course of a century of theory-focused, empirical research in behavioural science. This is both a strength and a weakness of the TDF. It is a weakness insofar as the depth of meaning of the domains may not be evident to researchers without training or experience in behavioural theory, so there is the potential for the TDF to be poorly or superficially applied. To use the framework effectively, researchers need to ‘dig deep’, beyond a superficial interpretation of the domains. Thus, interdisciplinary research teams using the TDF for the first time may benefit from the inclusion of a health psychologist on the team. On the other hand, a strength is that there is a wealth of evidence relating to these domains that can facilitate researchers to finesse their exploratory research and to map the results of TDF-based problem analysis on to intervention components. We would argue that this is an effective way to build a solid rationale for implementation interventions.

### Potential for further elaboration of the theoretical domains

Although the TDF was developed to represent theories of organisational behaviour as well as individual behaviour, some researchers interested in organisational-level factors may feel that such factors are not adequately elaborated in the framework. Domains that arguably focus on these levels are Environmental Context and Resources, Social Influences, Social/Professional Role and Identity, and Behavioural Regulation. Table2 presents some examples of constructs in these four domains in the TDF [1] that represent the team or organisational levels and shows that several organisational constructs are indeed represented. Furthermore, it may be helpful here to distinguish between the level at which an intervention may be delivered (*e.g.*, incentives offered by the government to general practitioners for recording blood pressure readings of people with diabetes) and the level at which evidence of its impact may be measured (individual general practitioners more frequently record blood pressure readings). Hence, there appears to be reasonable representation in the framework of organisational factors that are relevant to clinical behaviours, although it is likely that further elaboration would enhance the usefulness of the framework for researchers interested in organisational-level influences.

**Table 2:** **Constructs in four theoretical domains, illustrating individual, team, and organisational levels (based on construct allocations reported by Michie*et al***[[1]]**)**

**Domain**	**Level**		
	**Individual**	**Team**	**Organisation**
**Environmental Context and Resources**	Environmental stressors Person × environment interaction	Environmental stressors	Resources/material resources (availability and management)
**Social Influences**	Social support Social pressure	Leadership Social comparisons	Organisational climate/culture Change management
**Social/Professional Role and Identity**	Identity Professional identity	Professional boundaries/role Group/social identity	Organisational commitment
**Behavioural Regulation**	Goal/target setting Self-monitoring	Goal/target setting Self-monitoring	Goal/target setting Barriers and facilitators

Nonetheless, some organisational theories are not concerned with the clinical actions performed by healthcare workers in the process of delivering healthcare. For example, Karasek’s Job Control Model [26] focuses on outcomes such as burnout, staff turnover, job satisfaction, and the like. Although these outcomes are important issues in their own right and are arguably related to the quality of healthcare provided, they are different from the actions of healthcare workers as they deliver healthcare and so are beyond the scope of the TDF. Thus, not all organisational theories are appropriate for inclusion in the framework.

The domain Nature of the Behaviour is often omitted in TDF-based studies, with the reasoning that it relates more to an understanding of the characteristics of the behaviour itself than to influences on behaviour [13]. However, it may be worth considering the wealth of research following the Diffusion of Innovations approach [27] suggesting that the nature of the innovation (*i.e.*, the behaviour(s) targeted for change) can have a large impact on its adoption. Inclusion of key factors (*e.g.*, is the target behaviour complex, observable, trialable, high profile [28]) could render this domain highly relevant for designing interventions and predicting behaviour change.

### Links between the theoretical domains framework and other frameworks

The TDF is potentially compatible with a range of existing frameworks in the implementation literature. For example, Kitson and colleagues [29] called for the integration of theoretical perspectives into the Promoting Action on Research in Health Services (PARiHS) framework. The TDF could be useful in elaborating some components of the ‘diagnostic and evaluation’ stage of PARiHS. Damschroder and colleagues [30] proposed a Consolidated Framework for Implementation Research (CFIR); there is potential for mapping the TDF domains on to constructs in this framework (in particular, within Outer Setting, Inner Setting, and Characteristics of Individuals). The advantage of such a process would be to provide access to a large evidence base from the behaviour-change literature that could be useful in CFIR-based research.

### Further potential of the theoretical domains framework in implementation research

The TDF is amenable to application in study designs other than interview and questionnaire studies. For example, it may be used as a conceptual framework to guide structured observation of the target group and/or supplementary data collection from other stakeholders (that is, others affected by performance/nonperformance of behaviour, such as patients or colleagues). TDF-based interview and observational studies may thus be seen as hypothesis-generating investigations that are appropriate in the earlier, exploratory phases of an implementation research programme.

### The theoretical domains framework thematic series

In this TDF thematic series, a number of papers report exploratory research with respect to a range of clinical behaviours that, evidence suggests, should change so that gains can be made in quality of healthcare and patient outcomes. Other papers in the series illustrate methods that can be used when applying the TDF to problem analysis, intervention design, and process evaluation. Such approaches are consistent with calls for more systematic methods of developing interventions, specifying the ‘active ingredients’ of interventions and theorising the pathways to change [31,32].

The TDF was formulated to enhance the usefulness of behavioural theory to researchers in a range of disciplines. Its success in generating research and contributing to problem solving in implementation research could be seen as a confirmation of its usefulness. Nonetheless, further interrogation, validation, and refinement would seem appropriate. A key paper in this series reports a validation study that independently assesses the structure of the framework [33].

## Conclusion

The TDF describes a comprehensive range of potential mediators of behaviour change relating to clinical actions. It thus provides a useful conceptual basis for exploring implementation problems, designing implementation interventions to enhance healthcare practice, and understanding behaviour-change processes in the implementation of evidence-based care. Nonetheless, some unresolved issues remain. These include the relationships between the theoretical domains, possible lack of precision of boundaries between domains, how best to elaborate and operationalise the framework, how best to design interventions informed by TDF-based problem analysis, and how best to accumulate evidence to refine the content of the framework. There are opportunities for further international collaborative research to address these issues.

## Competing interests

DO is an Associate Editor of *Implementation Science*. All editorial decisions regarding this article and all subsequent articles in the TDF series were made independently by Robbie Foy, Deputy Editor-in-Chief.

## Authors’ contributions

JJF drafted the initial form and all revisions of this manuscript. JC and DO provided input, feedback, and substantial refinements. All authors agreed to the final manuscript.
